# PALLiative care in ONcology (PALLiON): A cluster-randomised trial investigating the effect of palliative care on the use of anticancer treatment at the end of life

**DOI:** 10.1177/02692163231222391

**Published:** 2024-01-09

**Authors:** Marianne Jensen Hjermstad, Aleksandra Pirnat, Nina Aass, Sigve Andersen, Guro L Astrup, Olav Dajani, Herish Garresori, Kristin V Guldhav, Hanne Hamre, Ellinor C Haukland, Frode Jordal, Tonje Lundeby, Erik Torbjorn Løhre, Svein Mjåland, Ørnulf Paulsen, Karin A Semb, Erik S Staff, Torunn Wester, Stein Kaasa

**Affiliations:** 1 Regional Advisory Unit in Palliative Care, Department of Oncology, Oslo University Hospital, Oslo, Norway; 2 European Palliative Care Research Centre (PRC), Department of Oncology, Oslo University Hospital, and Institute of Clinical Medicine, University of Oslo, Oslo, Norway; 3 Institute of Clinical Medicine, University of Oslo, Oslo, Norway; 4 Department of Oncology, University Hospital of North Norway, Tromsø, Norway; 5 Institute of Clinical Medicine, UiT, The Arctic University of Norway, Tromsø, Norway; 6 Department of Hematology and Oncology, Stavanger University Hospital, Stavanger, Norway; 7 Department of Oncology and Palliative Care, Førde Hospital Trust, Førde, Norway; 8 Department of Oncology, Akershus University Hospital, Akershus, Norway; 9 Department of Oncology and Palliative Care, Nordland Hospital Trust, Nordland, Norway; 10 Department of Clinical Oncology, Østfold Hospital Trust, Østfold, Norway; 11 Cancer Clinic, St. Olavs hospital, Trondheim University Hospital, Trondheim, Norway; 12 Department of Clinical and Molecular Medicine, Faculty of Medicine and Health Sciences, NTNU, Norwegian University of Science and Technology, Trondheim, Norway; 13 Center for Cancer Treatment, Sorlandet Hospital, Kristiansand, Norway; 14 Palliative Care Unit, Telemark Hospital Trust, Skien, Norway; 15 Department of Oncology and Palliative Care, Vestfold Hospital Trust, Tønsberg, Norway; 16 Department of Oncology, Ålesund Hospital Trust, Ålesund, Norway

**Keywords:** Antineoplastic agents, end-of-life care, palliative care, patient-reported outcomes, randomised controlled trial

## Abstract

**Background:**

Effects on anticancer therapy following the integration of palliative care and oncology are rarely investigated. Thus, its potential effect is unknown.

**Aim:**

To investigate the effects of the complex intervention PALLiON versus usual care on end-of-life anticancer therapy.

**Design:**

Cluster-randomised controlled trial (RCT), registered at ClinicalTrials.gov (No. NCT01362816). The complex intervention consisted of a physician education program enhancing theoretical, clinical and communication skills, a patient-centred care pathway and patient symptom reporting prior to all consultations. Primary outcome was overall use, start and cessation of anticancer therapy in the last 3 months before death. Secondary outcomes were patient-reported outcomes. Mixed effects logistic regression models and Cox proportional hazard were used.

**Setting:**

A total of 12 Norwegian hospitals (03/2017–02/2021).

**Participants:**

Patients ⩾18 years, advanced stage solid tumour, starting last line of anticancer therapy, estimated life expectancy ⩽12 months.

**Results:**

A total of 616 (93%) patients were included (intervention: 309/control:307); 63% males, median age 69, 77% had gastrointestinal cancers. Median survival time from inclusion was 8 (IQR 3–14) and 7 months (IQR 3–12), and days between anticancer therapy start and death were 204 (90–378) and 168 (69–351) (intervention/control). Overall, 78 patients (13%) received anticancer therapy in the last month (intervention: 33 [11%]/control: 45 [15%]). No differences were found in patient-reported outcomes.

**Conclusion:**

We found no significant differences in the probability of receiving end-of-life anticancer therapy. The intervention did not have the desired effect. It was probably too general and too focussed on communication skills to exert a substantial influence on conventional clinical practice.

**What is already known about the topic?**

- Randomised controlled trials (RCT) show favourable patient-reported outcomes of specialised palliative care in several domains of quality of life.
- International guidelines do not recommend anticancer treatment at the end of life, as this may compromise quality of life, often with negligible antitumor effect.
- Still, this practice continues.

**What this paper adds?**

- The complex intervention of this RCT with education and palliative care including patient-reported outcome measures did not have the desired effect on the provision of anticancer treatment at end of life between the groups.
- The fidelity to the intervention was probably too low to change working behaviour, which requires a solid anchoring to gain momentum.

**Implications for practice, theory or policy**

- Introducing a change in clinical practice implies a profound impact in the way people work every day.
- A sustainable change of practice is difficult to achieve in the context of a clinical trial.
- We recommend the use of implementation science principles and strategies to increase the chances of success in changing working behaviour.

## Background

Evidence from randomised controlled trials (RCTs) confirm benefits of hospital-based specialised palliative care alongside anticancer therapy on multiple clinical and patient-centred outcomes in patients with advanced disease, as summarised in a Cochrane review.^1^ Although effect sizes were small, results were of high clinical relevance to patients. Twenty-one of 42 RCTs were in cancer samples and 11 had quality of life as the primary outcome.

It is well known that the intensity of all end-of-life anticancer therapy may have pronounced side effects and significantly impair patient well-being.^2,3^ This is particularly important when the tumour effects are marginal^3,4^ and accentuates the need to balance treatment intensity with palliative and patient-centred perspectives,^5^ focussing on the person with the cancer, including quality of life and care preferences.

Determining exactly when to discontinue anticancer therapy remains a clinical challenge and is influenced by treatment traditions, professional and attitudinal barriers and the societal demand for treatment and cure.^5^ The American Society of Oncology (ASCO) defines discontinuation of end-of-life chemotherapy as one factor that may improve the quality of care and does not recommend its use in the last month of life,^6^ aligned with the ESMO clinical practice guidelines.^7^ Registry-based studies have shown that close to 20% received anticancer therapy in the last two weeks of life,^8^ most often in centres with no specialised palliative care units.^4,9^

No RCTs on integration of oncology and specialised palliative care use anticancer therapy as the primary endpoint, despite reported associations with poorer quality of life and hastened death^2^ and although indices of less use have been reported as secondary outcomes.^10,11^ Most publications on end-of-life anticancer therapy are retrospective cohort analyses from single- or multi-institutional studies^2,9,12,13^ or registry reports^8,14–16^ suggesting that palliative care and patient-centred care may reduce aggressive end-of-life treatment.

To this end, we developed the cluster-RCT PALLiON (PALLiative care integrated in ONcology) with a complex intervention of an educational program and a patient-centred care pathway alongside anticancer therapy versus usual care. Our hypothesis was that the complex intervention would result in less use of end-of-life anticancer therapy, based on a better understanding of the non-beneficial effects at this stage.^2^ The primary study outcome was overall use, start and cessation of anticancer therapy in the last 3 months before death. Secondary outcomes were patient-reported symptoms and quality of life.

## Methods

### Design

A national, parallel group, cluster-RCT. The study protocol has been published previously.^17^

### Setting

**Figure 1.**
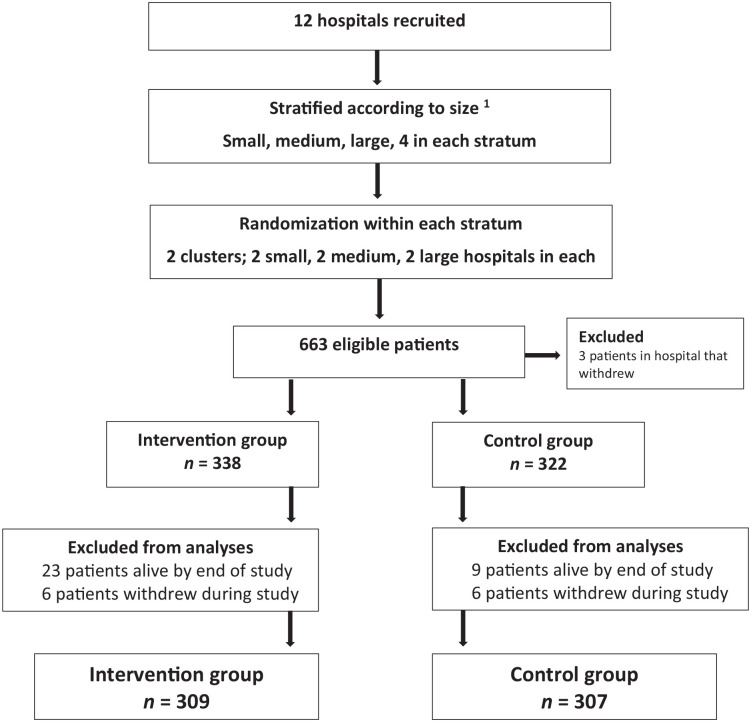
The trial profile.

PALLiON was performed in 12 Norwegian cancer centres, all with a well-defined local catchment area for anticancer therapy with established oncology and specialised palliative care programs. The hospitals were grouped into three strata, by size of the catchment areas: small, medium and large, Figure 1. One hospital in the control arm withdrew shortly after study start and was replaced by a new hospital within the same stratum.

### Randomisation

Stratified, blinded randomisation (web-based) was performed within each stratum to decrease the chance of cluster imbalance. Blinding at the participant level was not relevant.

### Intervention

The composition of the complex intervention was informed by the literature on integration of specialised palliative care and oncology, patient-centred care, anticancer therapy use, end-of-life care and treatment and supplemented with communication science studies. Previous RCTs have used hospital-based, multi-professional specialised palliative care, with a wide variety of interventions and assessment of patient-reported outcomes.^1^ We wanted to develop an intervention that could aid in reducing the excessive use of anticancer therapy with a stronger involvement of the oncologists.

**Figure 2.**
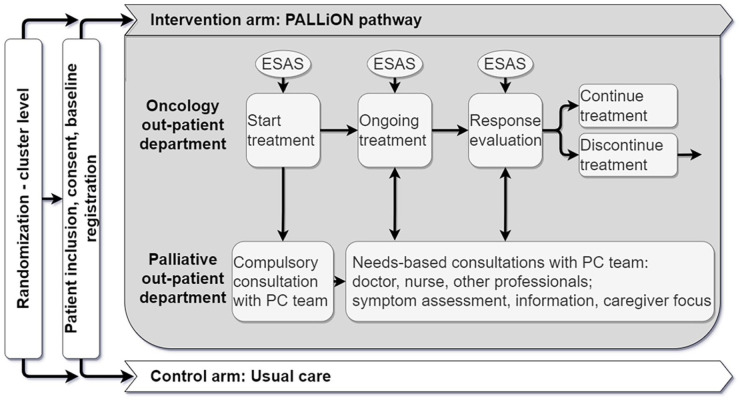
The PALLiON care pathway during anticancer treatment.

The intervention comprised:

(1) An educational program for physicians (oncologists, MDs in specialist training) conducted prior to patient inclusion aiming to enhance their theoretical, clinical and communication skills to appreciate the benefits of integrated oncology and palliative care.^18^ This target group was chosen given their formal and informal position to influence clinical decisions. The principal investigators at each hospital were responsible for the education of the local multi-professional PALLiON teams, supported by the PALLiON leader team. The program encompassed lectures, an e-learning program and skills training and coaching that has been thoroughly described^18^ (Supplemental Table 1). The lectures’ contents corresponds with the organisation of care and the different steps of the patient-centred care pathway (described below) and includes training in skills that are often perceived as difficult by clinicians, such as communication of prognosis and breaking bad news.
(2) A PALLiON-specific patient-centred care pathway, Figure 2, purposefully chosen as a recommended model.^5^ All consultations were in-person. The oncologists were responsible for the anticancer therapy and the specialised palliative care-teams for the palliative care, with collaboration on patient-centred issues and focussing on shared decision-making. The pathway encompassed:
• Mandatory referral to specialised palliative care for all patients upon start of anticancer therapy
• Documentation of tumour-centred and patient-centred data in the patient records and the use of these, for example, performance status, weight loss and patient preferences to decide initiation or cessation of anticancer therapy
• Registration of symptoms using the Edmonton Symptom Assessment System (ESAS)^19^ or a digital tool, Eir,^20^ prior to all consultations (numerical scales 0–10), and systematically discussing scores with the patients, with subsequent documentation
• PALLiON templates in the patient records to advise the agenda of the consultations and to document main issues addressed^17^

### Participants

Inclusion criteria were age ⩾18 years, residing in the hospitals’ catchment area, cognitively capable of providing written consent, metastatic or advanced solid tumours, estimated life expectancy of ⩽12 months and coming for start of the anticipated last line of anticancer therapy (03/2017–02/2021), according to Norwegian consensus-based treatment guidelines. Exclusion criteria were inability to provide consent, and lung, gynaecological and haematological cancer as these diagnoses are not treated in the general oncology departments throughout Norway.

The control arm followed usual care regarding anticancer therapy (following treatment guidelines), palliative care referrals and collaboration between oncologists and specialised palliative care teams.

### Data collection

Clinical and treatment related data, Supplemental Table 2, were registered every other month from inclusion up to 1 year or until death/study closure on a web-based case report form.^17^

All patients received the EORTC Quality of Life Questionnaire Core 15 Palliative (QLQ-C15-PAL)^21^ by mail every other month from inclusion up to 1 year. This well-validated tool contains relevant symptoms in palliative care, that is, physical and emotional function, pain, fatigue, dyspnoea, sleep, constipation, appetite loss, nausea/vomiting and Global quality of life, scored as 1–4 (*not at all-very much*) or 1–7 (*very poor-excellent*), and transformed to 0–100 scales.

### Outcome measures

Primary outcome was overall use of anticancer therapy and defined as administration of conventional chemotherapy, immunotherapy and targeted therapies, as in other studies.^10,11^ Secondary outcomes were QLQ-C15-PAL scores.

### Statistical analyses

Sample size calculation (300 patients per arm) was based on the primary outcome; use of anticancer therapy in the last 3 months of life,^17^ accounting for hospital clustering, Supplemental Table 3.

Given the multilevel structure clustering of the data and binary outcome variable (anticancer therapy use yes/no), mixed effects logistic regression was employed using fixed factors age, sex, diagnosis, Karnofsky Performance Status at inclusion and a random intercept for the cluster level. A maximum likelihood estimation method was chosen as the risk of bias is negligible with a sample of this size. An additional model with exclusion of performance status was examined to explore if a potential change in performance status over time would affect the main outcome. Further, another three models were tested to examine a potential influence of three QLQ-C15-PAL scales (Global quality of life, physical function, fatigue) at the time of inclusion instead of performance status. These models included three of the most frequently used scales in prognostic analyses.^22^

Cox proportional hazard method was used to model time and survival curve from the end of last cycle to death, adjusted for age, sex, diagnosis, performance status at inclusion and hospital size, but did not account for hospital clustering. As the administration of immunotherapy and targeted therapies as anticancer therapy differs from guidelines for conventional chemotherapy and the limited use of these modalities in PALLiON,^17^ the Cox analyses did not include these. A 10% change in the QLQ-C15-PAL scores was used to indicate a clinically significant difference. SPSS v28.0 (IBM Corp. Armonk, NY) was used.

### Ethical approval and consent

All patients provided written informed consent. The study was approved by the Regional Committee for Medical and Health Research Ethics, South-East Norway (2016/1220-PALLiON), the Data Protection Official at OUS and the hospitals’ Institutional Review Boards and registered at ClinicalTrials.gov (No. NCT01362816) in March 2017. No particular ethical issues were raised.

## Results

### Patient characteristics

Overall, 663 patients were included. Forty-seven were excluded prior to analyses, Figure 1, leaving 616 (93%) for analyses; intervention: 309, control: 307. Median age was 69 (IQR 62–75), and gastrointestinal cancers dominated (77%). Median survival time from inclusion to death was 8 (IQR 3–14) and 7 months (IQR 3–12) (intervention/control). More intervention patients used non-opioids (37% versus 23%, *p* < 0.001, Table 1).

**Table 1.** Patient characteristics, overall and by group.

Characteristics	Total (*N* = 616)	Intervention (*n* = 309)	Control (*n* = 307)	*p* Value
Patients included by hospital size (%)	*n* (%)			
Large	326 (53)	164 (53)	162 (53)	
Medium	179 (29)	85 (28)	94 (31)	0.55
Small	111 (18)	60 (19)	51 (17)	
Age	Median (IQR)			
	69 (62–75)	68 (61–75)	70 (62–75)	0.42
	*n* (%)			
Male sex	389 (63)	193 (62)	196 (64)	0.74
Karnofsky performance status score				0.49
100–80	438 (71)	224 (72)	214 (70)	
70–50	174 (28)	84 (27)	90 (29)	
40–0	4 (1)	1 (0)	3 (1)	
Comorbidities				
Yes	313 (51)	164 (53)	149 (49)	0.24
Primary cancer diagnosis				0.05
Gastrointestinal	475 (77)	251 (81)	215 (70)	
Prostate	40 (6)	22 (7)	18 (6)	
Breast	29 (5)	10 (3)	19 (6)	
Melanoma	20 (3)	5 (2)	15 (5)	
Urothelial	14 (2)	5 (2)	9 (3)	
Renal	9 (1)	3 (1)	6 (2)	
Other	29 (5)	13 (4)	16 (5)	
Metastases				
Yes	572 (93)	283 (92)	289 (94)	0.27
Prior anticancer therapy				
Yes	369 (60)	185 (60)	184 (60)	0.99
Prior anticancer therapy modalities				
Systemic anticancer therapy	91 (15)	51 (17)	40 (13)	0.38
Surgery	50 (8)	20 (6)	30 (10)	0.17
Radiotherapy	8 (1)	3 (1)	5 (2)	0.47
Combined treatments	220 (36)	111 (36)	109 (36)	0.45
Analgesic medication at inclusion				
Opioids	192 (31)	102 (33)	90 (29)	0.33
Non-opioid analgesics	185 (30)	114 (37)	71 (23)	<0.001
Opioid + non-opioid analgesics	106 (17)	65 (21)	41 (13)	<0.001
Place of death				
Hospital	228 (37)	101 (33)	127 (41)	
Nursing home	173 (28)	99 (32)	74 (24)	<0.001
At home	117 (19)	58 (19)	59 (19)	
Other	36 (6)	26 (8)	10 (3)	
Unknown	62 (10)	25 (8)	37 (12)	
Patients receiving anticancer therapy during study	603 (98)	300 (97)	303 (99)	0.32
Patients receiving anticancer therapy last 3 months	177 (29)	79 (26)	98 (32)	0.36
	Median (IQR)			
Days from start of chemotherapy^a^ to death (*n* = 546)	188 (77–370)	204 (90–378)	168 (69–351)	0.24

a Conventional chemotherapy, not including Immunotherapy and Targeted therapies

Median number of days between start of conventional chemotherapy and death was 188 (IQR 77–370) overall, intervention: 204 (IQR 88–378), control: 168 (69–351). Place of death varied between groups.

Thirteen patients with a short median survival time (<2 months) did not receive any anticancer therapy during the study.

### Use of anticancer therapy in the last 3 months

Overall, 177 (29%) patients received anticancer therapy in the last 3 months of life (intervention: 79 [45%], control: 98 [55%]). Median time from diagnosis until death was 6 months (IQR 3–22). The majority (35%) had pancreatic cancer.

### Conventional chemotherapy in the last 3 months

Of the 177, 151 (85%) patients started a chemotherapy cycle in the last 3 months of life, at a median of 44 days (IQR 32–61) before death and discontinued at a median of 39 days (IQR 28–57) before death. Ninety-six started chemotherapy at a median of 36 (IQR 25–42) days and stopped at a median of 33 days (IQR 21–41) before death. Thirty patients started chemotherapy at a median of 18 days (IQR 12–25), with discontinuation at a median of 15 (IQR 10–21) days before death. Breakdown per group is reported in Table 2.

**Table 2.** Patients receiving chemotherapy during the last 3 months before death, and the proportion of patients who started and ended this treatment.

	Last 3 months		Last 2 months		Last month	
	Intervention	Control	Intervention	Control	Intervention	Control
Number of patients (%)^a^	79 (26)	98 (32)	59 (19)	67 (22)	33 (11)	45 (15)
Time since diagnosis						
Days before death, median	163	180	203	181	149	86
(IQR)	(102–536)	(85–783)	(94–520)	(70–790)	(62–324)	(61–709)
Most prevalent diagnoses *n* (%)						
Pancreatic	30 (38)	31 (32)	21 (36)	21 (31)	13 (39)	13 (29)
Colorectal	20 (25)	18 (18)	16 (27)	14 (21)	9 (27)	11 (24)
Starting chemotherapy^b^						
Number of patients	70	81	48	48	14	16
Days before death, median	46	42	35	36	19	16
(IQR)	(32–61)	(32–61)	(26–49)	(26–40)	(13–25)	(13–26)
Ending chemotherapy^>b,c^						
Number of patients	74	82	53	54	18	17
Days before death, median	37	40	33	36	16	15
(IQR)	(25–58)	(32–55)	(19–42)	(25–40)	(10–19)	(13–21)

a Number and percentage of patients receiving chemotherapy by group: intervention *n* = 309, control *n* = 307.

b Not Including immunotherapy and targeted therapies.

c Number of patients ending chemotherapy included 5 patients who had started a cycle shortly before the last 3 months, and who ended the treatment within 3 months before death.

### Immunotherapy and targeted therapies in the last 3 months

Thirteen patients (urological [*n* = 7], melanomas, colorectal and breast cancers [all *n* = 2]) received immunotherapy/targeted therapies in the last 3 months of life, starting at a median of 35 days before death (IQR 25–45) for intervention and 53 days (IQR 39–67) for control.

### Anticancer therapy and time between cessation and death

The mixed effects logistic regression model showed no significant differences between groups in the probability of receiving anticancer therapy at any assessment point. The odds ratios (OR) in the intervention versus control group were: OR 0.64 (95% CI 0.19–2.08; *p* = 0.46); last 2 months OR 0.78 (95% CI 0.21–2.94; *p* = 0.71), the last month OR 0.99 (95% CI 0.17–5.72; *p* = 0.99). No significant differences were found between strata, neither within nor between the clusters in the explored mixed effects model (*p* = 0.56). The same applied to a model that excluded performance status as a fixed effect, nor when replacing performance status with the QLQ-C15-PAL scales (Global quality of life, physical function or fatigue) in additional models.

**Figure 3.**
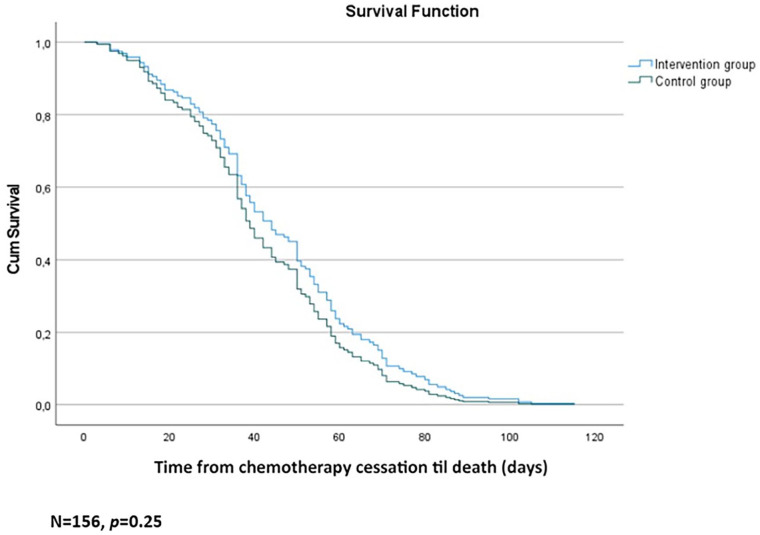
Adjusted survival curve (Cox model) for time from chemotherapy cessation and death during the last 3 months of life) by groups. *N* = 156, *p* = 0.25.

The adjusted Cox survival model, Figure 3, showed a longer time interval between chemotherapy cessation and death in the intervention group, albeit non-significant (*p* = 0.25; HR = 0.81 95% CI 0.57–1.15), and for patients with high performance status at inclusion (*p* < 0.001).

### Patient reported outcomes

Five hundred and sixty-two patients (91%) completed at least one questionnaire. No statistically or clinically significant differences were found between groups at inclusion, Supplemental Table 4. Two-hundred and fifteen patients (38%) had complete questionnaires at inclusion, 2, 4 and 6 months. The only statistically significant differences from inclusion to 6 months were a deterioration in physical function and less sleep problems and constipation, albeit not clinically significant, that is, exceeding 10% (Table 3). The median interval between last questionnaire and death was almost the same (intervention: 8.5 weeks [IQR 5–17], control: 7 weeks [IQR 4–14]). Supplemental Figures 1a–c display mean scores over time for Global quality of life, physical function and fatigue for patients grouped according to attrition over the first 6 months.

**Table 3.** Differences in EORTC QLQ-C15-PAL scores from inclusion to 6 months for patients with complete assessments (*n* = 215).

EORTC QLQ-C15-PAL scores	Inclusion	Month 6	Test statistics		
	*M* (*SD*)	*M* (*SD*)	*df*	*T*	*p* ^a^
Global quality of life^b^	60.0 (23.7)	61.9 (25.2)	211	−0.83	0.41
Physical function^b^	80.9 (16.2)	76.4 (22.0)	210	3.00	**0.01**
Emotional function^b^	81.1 (20.7)	84.0 (19.9)	212	−1.96	0.05
Fatigue^c^	37.9 (24.4)	39.6 (24.5)	212	−0.88	0.38
Nausea /vomiting^c^	16.1 (23.5)	15.8 (23.1)	210	0.18	0.85
Pain^c^	28.9 (26.4)	25.8 (24.7)	212	1.34	0.18
Dyspnoea^c^	21.4 (25.5)	22.6 (27.0)	210	−0.58	0.56
Sleep^c^	27.5 (27.3)	22.3 (26.5)	211	2.34	**0.02**
Appetite loss^c^	28.6 (30.7)	25.2 (31.9)	2111	1.50	0.14
Constipation^c^	23.5 (29.2)	18.5 (24.7)	212	2.18	**0.03**

EORTC QLQ-C15-PAL = European Organisation for Research and Treatment of Cancer Quality of Life Questionnaire C15 Palliative^21^; *SD* = Standard deviation.

a Statistically significant *p*-values are highlighted in bold.

b Higher scores indicate better quality of life and function.

c Higher scores indicate higher symptom intensity.

## Discussion

### Main findings

This cluster-RCT using a complex intervention with education, and the PALLiON pathway with compulsory referral to palliative care, and regular assessment of patient-reported outcomes did not result in less anticancer therapy at end of life. Overall, 29% of the 616 patients who died during study received anticancer therapy in the last 3 months before death, declining to 13% in the last month.

No significant differences in the probability of receiving end-of-life anticancer therapy were found with age or sex, contrasting reports of more intensive end-of-life therapy in younger patients.^8,14^ The high proportion of advanced gastrointestinal cancer patients, <5% with last stage breast cancer and no lung, gynaecological and haematological patients, groups with anticancer therapy rates >20% in the last month of life,^2,8,11,15^ may explain this. There were no significant differences in end-of-life anticancer therapy use between the three stratification levels, contrasting reports of more intensive end-of-life treatment in university hospitals than in smaller hospitals.^8,23^ Norway’s public health care system ensures cancer treatment according to national anticancer therapy guidelines with negligible patient cost and has few private for-profit clinics.

### Who received anticancer therapy?

Treatment guidelines for patients with solid tumours^6,7^ recommend anticancer therapy only for those in good performance status (ECOG 0–1, or maybe ECOG 2), despite associations with poorer quality of life near death also in these patients.^2^ We found that patients with higher performance status at inclusion received chemotherapy significantly closer to death. This may reflect a poor prognosis from diagnosis for many patients in our study. Thus, a broader spectrum of diagnoses might have influenced these percentages as studies report substantial variations in end-of-life anticancer therapy across cancer diagnoses.^2,8,12,15^ The adjusted Cox survival model indicated a longer, non-significant interval between anticancer therapy cessation and death in the intervention group, with a similar distribution of diagnoses in the groups. Whether this is attributable to the intervention per se, cannot be concluded from our data.

Overall, 13% of our patients received anticancer therapy in the last month of life, comparable to 10% in a former Norwegian single centre study.^12^ These rates are lower than the 20% and 24% reported from Finnish^14^ and Italian^13^ centres, and the 23% in a secondary analysis from an Italian RCT on quality of life,^10^ but more in line with regional registry data in Italy of 15%.^15^ These results, however, contrast an anticancer therapy rate of 3.2% in a Norwegian retrospective registry-study with more than 52,000 patients.^16^ Differing rates and different samples under study may indicate less generalisability between countries.

Direct comparisons between studies that span several years, even decades, are difficult, and the use of immunotherapy/targeted therapies has received little if any attention in most studies. Canavan et al.^24^ reported a substantial change in the usage pattern of anticancer therapy in the US. The overall rate within 1 month of death remained stable at 39% over a 4-year period (2015–2019), but with a 10% decrease in chemotherapy counterbalanced with a similar increase in immunotherapy/targeted therapies.

The lack of an intervention effect on patient-reported outcomes contrasts with several previous RCTs^1^ but corresponds with another.^25^ This is probably attributed to patient-reported outcomes being the primary study outcome with associated power estimations in the positive RCTs. We found no clinically significant deterioration from inclusion to 6 months, probably reflecting a healthy bias.

### The missing effect of the complex intervention

A number of reasons may explain why the intervention was unsuccessful. The generally low national rates of end-of-life anticancer therapy, a strong adherence to national treatment guidelines, universal health coverage and few private facilities offering anticancer therapy indicate a more uniform treatment practice. Also, we do not know to what extent patients in the control group received specialised palliative care services, as this was by the discretion of the attending physician according to routine care. Hence, a substantial reduction in anticancer therapy following the intervention was probably too optimistic.

A reduction in anticancer therapy implies a profound change of practice, even in the context of a study. Doing this in a subset of the patients seen in an outpatient clinic, requires a high fidelity to study procedures and firm anchoring throughout the study, in which we may have fallen short. This was indicated in our paper describing a process analysis using a set of indicators for program fulfilment.^26^ The use of basic principles from implementation research, primarily a better up-front anchoring among health care providers and leaders and more emphasis on fostering engagement and fidelity to the intervention might have been profitable. Following this, we may have underestimated the need for continuous, almost daily, follow-up at all intervention sites, to manifest the outputs of the educational program and secure adherence to the PALLiON pathway. Despite being easily accessible by one click at all hospital computers, a process evaluation in one of the large hospitals revealed that most physicians did not use the consultation templates as intended.^26^

Apparently, the educational program did not have enough impact to change clinical practice, and the content’s applicability to reduce anticancer therapy can be discussed, especially given the strong emphasis on communication skills training and coaching. However, different elements in complex interventions act both individually and in combinations, with fidelity to the procedures being crucial. Also, the organisation was challenging, in terms of recruitment, attendance and individual follow-up. Most attendees however, perceived the program useful, in particular the younger,^18^ who were not in a position to change practice. It proved difficult to recruit senior doctors who constitute an influential group, meaning that a barrier for change was present already before patient inclusion.

Even if the effect sizes of patient-centred benefits in the integration RCTs are low, they are important to patients and to the quality of cancer care.^1^ Notwithstanding, we have not identified reports that present sustainable changes in the clinical organisation following this. PALLiON was initiated by the specialised palliative care teams, thus may have had less appeal in a larger tumour-focussed setting, a common barrier. The most obvious explanation for the lack of integration as we see it, is a need for substantial change of practice at multiple levels. Integration interferes directly with the way people work, and challenges physicians’ professional autonomy and perceptions of self-efficacy to manage palliative care needs.^27^ The increasing societal and political demands for anticancer therapy, enforced by technological and financial incentives counteract such changes.

Only a few negative papers from integration trials have been published; focussing on home care,^28^ improved self-reported problems,^29^ and effects on distress and quality of life.^25^ In the latter, Eychmuller et al.^25^ concluded that mutual understanding and interaction between oncology and palliative care are essential components for success, as addressed above. We believe that negative studies are worth publishing to advance reflections on clinical practice.

### Strengths and limitations

A study strength is using end-of-life anticancer therapy as the primary outcome. Some earlier integration studies also report on this, and other indices of aggressive end-of-life care, but most commonly as secondary outcomes or secondary analyses.^10,11^ Most data on end-of-life anticancer therapy come from retrospective cohort studies or registries providing important overviews, but not necessarily corresponding with clinical practice. We regard the cluster-randomised design a strength as clusters reduce the contamination effect and the stratification efficiently handles differences in hospital size. The more than 600 patients in the present study exceed the samples in the other four cluster-RCTs on integration that we have identified.^10,30–32^ However, the large confidence intervals suggest that study precision was low. Therefore, we cannot rule out negative nor positive effects, although none were identified.

The main limitation is the apparent insufficiency of the educational program to influence clinical practice. As corrections for multiplicity were not applied for secondary outcomes, conclusions about effects cannot be drawn, also applying to identifying small differences. The generalisability of our results may be limited as PALLiON was a national study. We believe that a department-wide, fully anchored integration program, drawing on continuous follow-up and implementation strategies among multi-professional personnel will improve the approach to anticancer therapy decisions at end of life, and better patient-centred care.

## Conclusion

The intervention had no effect on the anticancer therapy use at end of life. This may be due to a relatively low national use in Norway. Also, the intervention was too general and probably too focussed on communication skills to exert a substantial influence on traditional clinical practice. We recommend using implementation science strategies to succeed with changing work behaviour.

## Supplemental Material

sj-pdf-1-pmj-10.1177_02692163231222391 – Supplemental material for PALLiative care in ONcology (PALLiON): A cluster-randomised trial investigating the effect of palliative care on the use of anticancer treatment at the end of life Click here for additional data file. Supplemental material, sj-pdf-1-pmj-10.1177_02692163231222391 for PALLiative care in ONcology (PALLiON): A cluster-randomised trial investigating the effect of palliative care on the use of anticancer treatment at the end of life by Marianne Jensen Hjermstad, Aleksandra Pirnat, Nina Aass, Sigve Andersen, Guro L Astrup, Olav Dajani, Herish Garresori, Kristin V Guldhav, Hanne Hamre, Ellinor C Haukland, Frode Jordal, Tonje Lundeby, Erik Torbjorn Løhre, Svein Mjåland, Ørnulf Paulsen, Karin A Semb, Erik S Staff, Torunn Wester and Stein Kaasa in Palliative Medicine
