# Supplementary material for: PALLiative care in ONcology (PALLiON): A cluster-randomised trial investigating the effect of palliative care on the use of anticancer treatment at the end of life
Source: Palliat Med. 2024 Jan 9;38(2):229–39. doi: 10.1177/02692163231222391 (PMC10865754; doi:10.1177/02692163231222391)
Supplement: sj-pdf-1-pmj-10.1177_02692163231222391 – Supplemental material for PALLiative care in ONcology (PALLiON): A cluster-randomised trial investigating the effect of palliative care on the use of anticancer treatment at the end of life [file sj-pdf-1-pmj-10.1177_02692163231222391.pdf]

Supplemental Table 1. Overview of the educational program [1]

<table>
<tr><th>Lectures – content and format, total 3.5 hours</th><th>E-learning program – total 3.5 hours</th><th>Skills training and coaching</th></tr>
<tr><td>• PPT-presentations, taped, with voice-over for use in the E-learning program
• 7 lectures, 25-30 minutes, given by professors/oncologists/PC physicians</td><td>• The trajectory of Maria, age 38, metastatic breast cancer, followed when coming to the oncologist after a 4 week pause of line 3 of ACT until she approaches death.
• 4 videos of the doctor-patient encounter, 10-15 minutes, each presenting one or a few of the issues below.</td><td rowspan="8">Communication skill exercises in small groups led by pairs; one psychologist and one physician from PALLION.

Content of training reflects E-learning and topics brought up by participants in focus groups with oncologists/palliative care physicians/oncology and palliative care nurses.

Coaching: Real-life practice with one-to-one coaching sessions with each physician, following a PALLiON-specific coaching guide.</td></tr>
<tr><td>1 Presentation of PALLiON
• Care pathways
• Integration of PC and oncology</td><td>Film no. 1 illustrates how to:
• Set an agenda for the consultation
• Assess symptoms, including use of ESAS/Eir
• Introduce palliative care
• Elicit the patient voice</td></tr>
<tr><td>2 Presentation of the education program
• Objectives, structure, content and mode of working
• Short info about Eir</td><td>Film no. 2 illustrates how to:
• Communicate serious news
• Communicate prognosis at start of last line of ACT
• Communicate treatment intention and stop criteria
• Prepare patient and family for upcoming cessation of ACT</td></tr>
<tr><td>3 Using Eir in PALLiON
• Practical use
• Symptom assessment and treatment, general aspects</td><td>Film no. 3 illustrates how to:
• Talk about the termination of systemic ACT, “When is enough, enough?” (3 versions of the film)</td></tr>
<tr><td>4 Prognostication, prognostic tools
• Content and how to use the tools in clinical practice
• Communicating prognostic information to patients</td><td rowspan="4">Film no. 4 illustrates how to:
• Talk with the patient about the last phase of life and dying
• Focus on the family

The E-learning program also contains exercises designed to reflect on the contents of the films, learning objectives, presentations of specific communication skills and references to relevant literature.

Physicians are recommended to work with reflection exercises in pairs or groups.</td></tr>
<tr><td>5 Palliative radiotherapy
• General presentation of indications, fractionation regimes, effects, and side effects</td></tr>
<tr><td>6 Palliative ACT
• Presentation of indications, effects and side effects
• Shared decision making, when to stop/terminate</td></tr>
<tr><td>7 End-of-life
• General presentation of disease trajectory and anticipated symptoms during the last weeks
• The terminal phase</td></tr>
</table>

Ref: Lundeby et al. [18]

Supplemental Table 2. Sociodemographic information and clinical data in PALLiON, selected variables

Collected information	
**Basic background information**	**Time of registration**
Study specific information, Study ID, date of informed consent	Inclusion, collected from patient, family, electronic medical records
Ethnicity	
Level of education	
Living situation, housing	
Living situation, with/without others	
Work situation	
Smoking habits	
Alcohol consumption	
Comorbid conditions	
Height	
Weight	
Regular nutritional intake	
Cognition (date, time, day, backwards spelling)	
**Clinical data**	
Primary cancer diagnosis, date and stage	
Site and number of metastases	
Karnofsky Performance Status (KPS) score	
Regular medication	
Analgesics, more detailed	
Prior Anticancer Treatment	
**Clinical data related to the primary study outcome**	Every other month from inclusion until death/discontinuation. Collected from the electronic medical records
Anticancer treatment [1]	
Agents	
Start and cessation of chemotherapy cycles	
Medication use	
Use and dosage of opioids	
Use of additional analgesics	
Selected biomarkers	
Use of health care resources	
Primary care, hospitalization, emergency room visits, admissions nursing homes	
Imaging (MR/CT/PET)	
Transfusions	
Place of death	As applicable

[1] Including immunotherapy and targeted therapies

Supplemental Table 3. Details about the statistical work-up

Determination of a clinically relevant difference of 25% was based on a review of patient records at the largest intervention hospital (Oslo University Hospital, OUH), clinical experience, and previously published data. Variability in cluster size was calculated according to Van Breukelen [17]. A sample size of 300 patients in each arm accounts for ICC up to 0.08, while 340 patients per arm accounts for a 12 % attrition rate. The clustersampsi module in Stata was used (Stata Statistical Software, Release 14, College Station, TX, USA).
Standard descriptive statistics were used. Differences between the groups were analyzed using chi square, T-tests, median and Mann-Whitney tests, as appropriate. ACT use was explored by the intention-to-treat approach, while time estimations from treatment until death were performed for those receiving chemotherapy.
A complete description of the statistical reasoning and methods appears in the PALLiON protocol [17]

Supplemental Table 4. Differences in EORTC QLQ-C15-PAL scores between treatment groups at inclusion

EORTC QLQ-C15-PAL scores	All patients (*n*=544)	Intervention (*n*=274)	Control (*n*=270)	Test statistics		
	Mean (SD)	Mean (SD)	Mean (SD)	df	T	*p*
Global Quality of life [1]	56.5 (25.1)	56.3 (24.5)	56.6 (25.6)	540	-0.16	0.87
Physical function [1]	75.6 (19.9)	76.0 (20.1)	75.2 (19.7)	540	0.45	0.65
Emotional function [1]	79.1 (22.3)	78.8 (22.8)	79.4 (21.8)	541	-0.33	0.74
Fatigue [2]	44.6 (26.6)	43.3 (27.9)	45.9 (25.2)	540	-1.14	0.26
Nausea / vomiting [2]	19.3 (26.7)	18.0 (26.1)	20.6 (27.2)	538	-1.13	0.26
Pain [2]	33.0 (28.8)	32.5 (27.9)	33.4 (29.6)	541	-0.35	0.73
Dyspnea [2]	23.4 (27.3)	22.4 (26.9)	24.4 (27.6)	539	-0.88	0.38
Sleep [2]	30.6 (29.1)	29.9 (28.9)	31.3 (29.4)	538	-0.58	0.57
Appetite loss [2]	34.4 (32.9)	34.7 (32.7)	34.1 (33.1)	538	0.22	0.83
Constipation [2]	26.9 (31.3)	25.0 (30.5)	28.8 (32.0)	540	-1.40	0.16

Abbreviations: EORTC QLQ-C15-PAL = European Organisation for Research and Treatment of Cancer Quality of Life Questionnaire C15 Palliative [21]; SD = Standard deviation

[1] Higher scores indicate better quality of life and function

[2] Higher scores indicate higher symptom intensity

Supplemental Figure 1a. Mean scores, Global Quality of life [1] by number of responders per assessment point

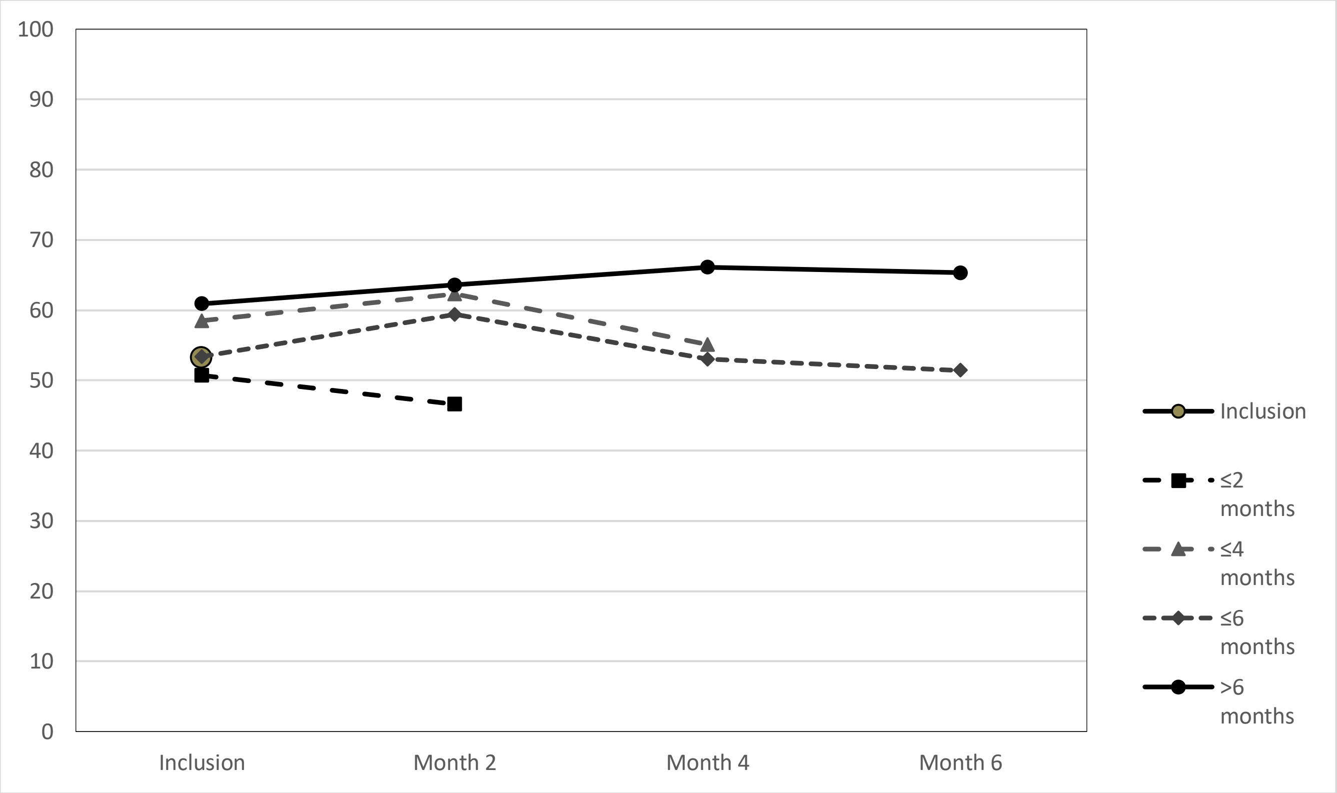

[1] EORTC QLQ-C15-PAL Global QoL Scale, higher score denotes better QoL [21]

Supplemental Figure 1b. Mean scores, Physical functioning [1], by number of responders per assessment point

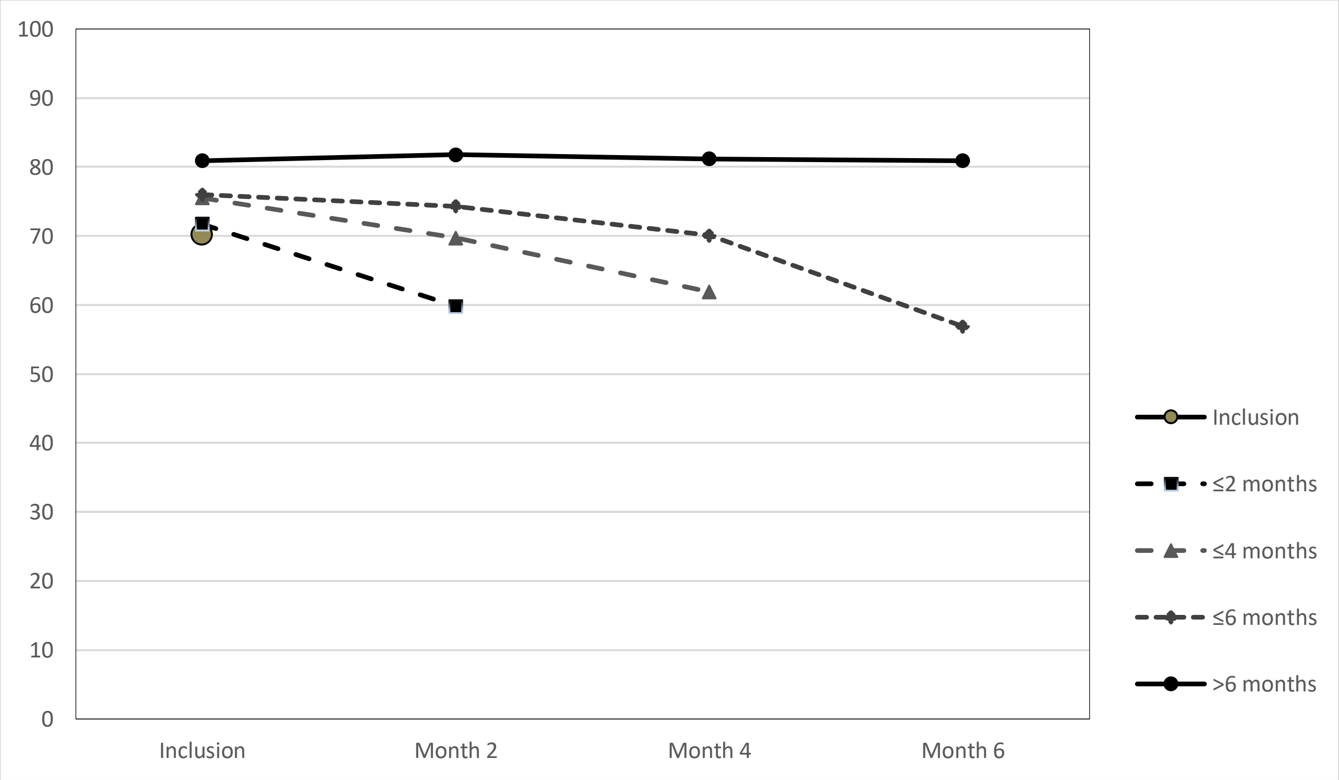

[1] EORTC QLQ-C15-PAL Physical Function Scale, higher score denotes better physical function[21]

Supplemental Figure 1c. Mean scores, Fatigue [1], by number of responders per assessment point

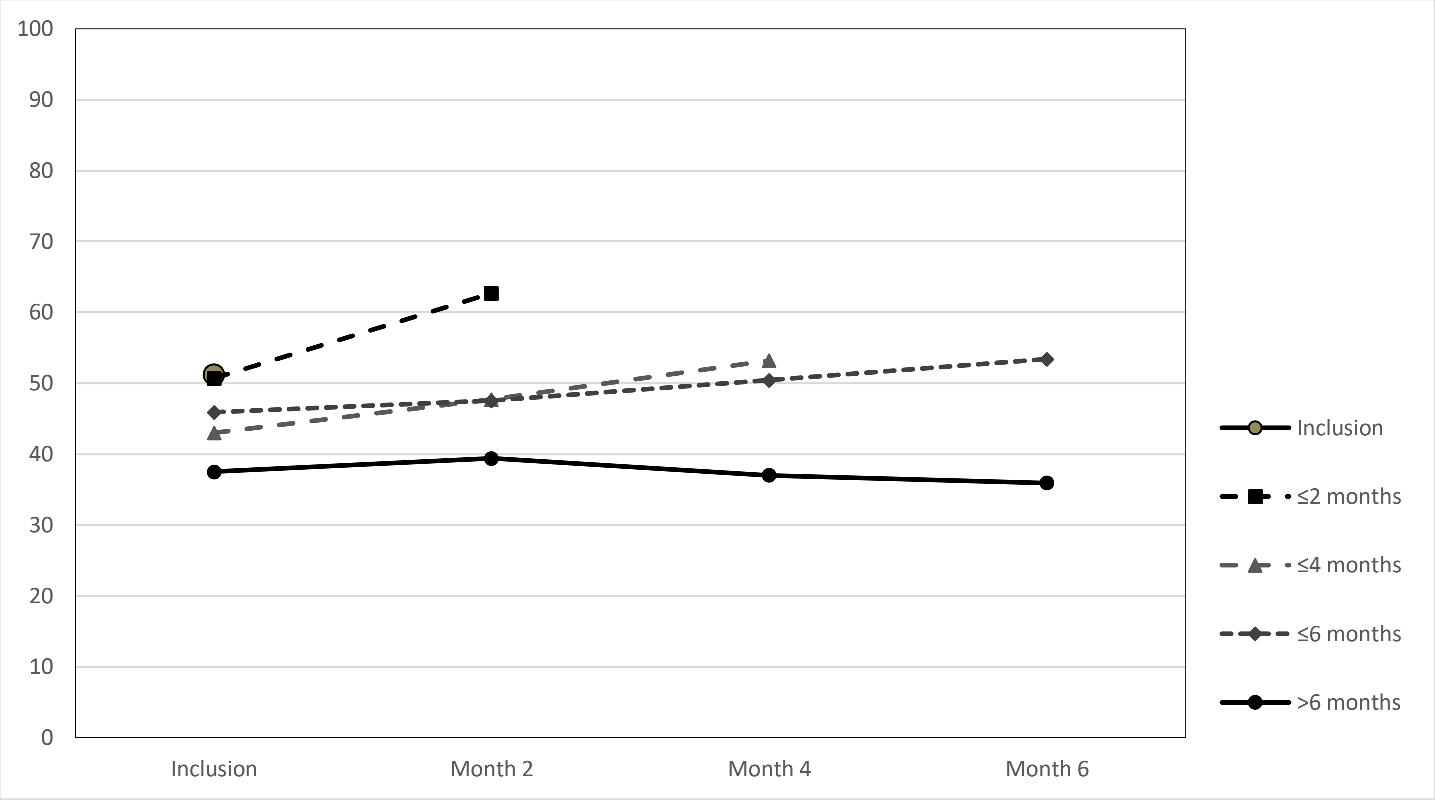

[1] EORTC QLQ-C15-PAL Fatigue scale, higher score denotes more fatigue[21]
